# Generating Localized Plasmonic Fields on an Integrated Photonic Platform using Tapered Couplers for Biosensing Applications

**DOI:** 10.1038/s41598-017-15675-0

**Published:** 2017-11-14

**Authors:** Gurpreet Singh, Renzhe Bi, U. S. Dinish, Malini Olivo

**Affiliations:** Laboratory of Bio-optical Imaging, Singapore Bioimaging Consortium, Agency for Science Technology and Research (A*STAR), Singapore, Singapore

## Abstract

A theoretical design and analysis of a tapered-coupler structure on a silicon nitride integrated-photonic platform for coupling optical energy from a dielectric waveguide to a plasmonic tip is presented. The proposed design can be considered as a hybrid photonic-plasmonic structure that generally supports hybrid symmetric and asymmetric modes. Along the taper, one of the hybrid modes approaches the cut-off, while the other approaches the short-range surface plasmon mode that generates localized fields. Potential use of the proposed novel tapered-coupler plasmonic structure for highly sensitive biosensing applications using surface enhanced Raman scattering (SERS) and metal enhanced fluorescence (MEF) techniques is discussed. For SERS, a theoretical electromagnetic enhancement factor as high as 1.23 × 10^6^ is deduced for taper tip widths as small as 20 nm. The proposed tapered-coupler sets up interesting possibilities towards moving to an all-integrated on-chip SERS and MEF based bio-sensor platform - away from traditional free-space based illumination strategies.

## Introduction

Plasmonics has been actively pursued over recent years to localize light into nanometer-scale regions with strong field-enhancements for a variety of applications that include data-storage^1,2^, biosensing^3^ and data-communications^4^. Several methods have been used to excite plasmonic fields and these methods can broadly be categorized into either free-space based methods, such as, using grating couplers, nanoantennas and prism couplers, or on-chip integrated-photonic based methods, such as, using optical waveguides^5–8^. Compared to free-space based methods, an on-chip approach can be advantageous to develop miniaturized systems that house both electronic and photonic components such as lasers, waveguides, sensors and detectors. Various coupling techniques have been developed for on-chip platforms to transfer optical energy from larger dielectric waveguides to nanometer-scale plasmonic structures. These coupling techniques can be based on either adiabatic^2^, resonant^6^ or a combination of both^7^ approaches. In resonant coupling, supermodes of hybrid waveguide structures interact among each other to beat the optical energy from a dielectric waveguide to a plasmonic waveguide. Resonant coupling can be sensitive to interaction lengths and mismatch between supermodes’ effective indices. In adiabatic coupling, a mode naturally transforms itself from one form to the other.

Recently, an adiabatic based tapered-coupler^2^ was proposed on an integrated silicon photonics platform, for data storage applications, to couple optical energy from a dielectric waveguide to a plasmonic tip with good coupling efficiency and strong localized fields within 20 nm tip width regions. In this report, we aim to further extend on the proposed adiabatic tapered-coupler on a silicon nitride (*Si*_3_ *N*_4_) platform for biosensing applications. Compared to complementary metal–oxide–semiconductor (CMOS) compatible materials such as silicon and silicon dioxide, silicon nitride is preferred due to a combination of higher refractive index than silicon dioxide (that allows for tighter light confinement and smaller device footprints), lower absorption losses at visible-to-near-infrared wavelengths than silicon (that allows for probing of biological media) and low background fluorescence^9,10^. In this report, we present a systematic design and analysis of a tapered-coupler on a silicon nitride platform that allows for coupling of optical energy from a dielectric waveguide to a plasmonic tip. The design analysis includes modal, coupling and resonance analysis of allowed modes within the tapered-coupler. The design and investigations in this paper are different from those proposed for the adiabatic based tapered-coupler^2^ in that (1) the design is specially for biomedical applications of SERS and MEF within the wavelength regime of 800 nm, (2) the design uses silicon nitride as the platform material and (3) further investigations are made to verify the resonance characteristics of the structure, the radiation enhancements and coupling efficiency of dipole oscillation at the tip of the structure into the fundamental waveguide mode.

An interesting application of the proposed plasmonic tapered-coupler is in biosensing. Surface enhanced Raman scattering (SERS)^11^, a versatile biosensing technique, relies on the giant enhancement of vibrational fingerprint Raman spectra of analyte molecules when it is absorbed onto nano-roughened metallic (gold/silver) surfaces. This giant enhancement is predominantly contributed by the plasmonics at sharp metallic tips through nanoscale surface engineering^12^. Among these, metallic nanotips and nanogaps have been used as the most sought-after geometry in achieving maximized SERS enhancements. Similarly, the growing interest in metal enhanced fluorescence (MEF) and its applications in biosensing and imaging has opened up the possibilities of using specially designed plasmonic nanostructures for enhancement of fluorescent dyes with low quantum yields^13–18^. As in the case of SERS, MEF also employs planar plasmonic substrates or metallic colloidal nanoparticles such as gold or silver^19,20^.

One of the limitations of such SERS and MEF based biosensors is that they require free-space illumination strategies that can be bulky and large. Moving towards an integrated on-chip platform based biosensor, where the excitation, sensing and detection sub-systems are all integrated on a chip, can allow for mobility, compactness and low-cost. Recently, Peyskens *et al*. demonstrated an integrated-photonic based approach to both excite and collect Raman emissions though a dielectric waveguide^21^. To enhance the Raman emission, metallic bow-tie nanoantennas were integrated on top of these dielectic waveguides. In this report, we discuss how our proposed tapered-coupler can be used as an elegant and alternative approach to both excite and collect Raman emissions through *Si*_3_ *N*_4_ waveguides.

## Results

**Figure 1:**
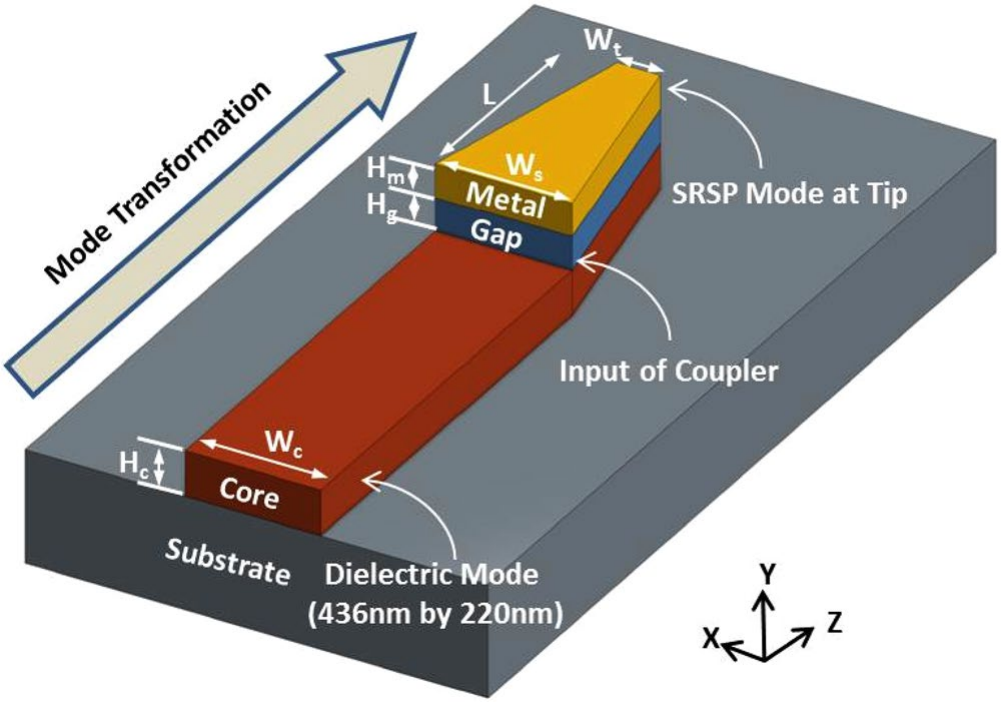
Schematic of proposed tapered-coupler on a *Si*_3_ *N*_4_ platform.

Figure 1 highlights the proposed tapered-coupler using a *Si*_3_ *N*_4_ waveguide core platform. The tapered-coupler is able to couple a fundamental TM mode from a *Si*_3_ *N*_4_ waveguide to a plasmon mode, with strong field intensities, at the tip of the coupler. As shown in Fig. 1, the proposed tapered-coupler can be considered as a hybrid waveguide consisting of a *Si*_3_ *N*_4_ core dielectric waveguide and a dielectric-metal-dielectric (DMD) waveguide placed on top, with gold (*Au*) as the metal. The spacer layer between the *Si*_3_ *N*_4_ dielectric core and the *Au* metal is silicon dioxide (*SiO*_2_). The hybrid waveguide has an initial width (*W*_*s*_) of 436 nm which is tapered down to a tip width (*W*_*t*_) of 50 nm over a length (*L*) of 425 nm. 50 nm has been selected as the tip width to accommodate potential fabrication limitations. The refractive indices of *Si*_3_ *N*_4_, *SiO*_2_ and *Au* are 1.996, 1.45, and 0.14 - j4.541^22^, respectively. The wavelength of interest is around 800 nm. The height of the *Si*_3_ *N*_4_ core is selected as 220 nm - a typical height for *Si*_3_ *N*_4_ based waveguides^23^. The height of the *SiO*_2_ spacer and *Au* metal layers are selected as 20 and 30 nm, respectively. A relatively thin layer of metal is selected to facilitate light coupling to the top metal surface, that forms the desired plasmonic mode^2^. The substrate is assumed as silicon dioxide and the cladding is assumed as a dielectric (water) with refractive index of 1.326.

### Mode Analysis

**Figure 2:**
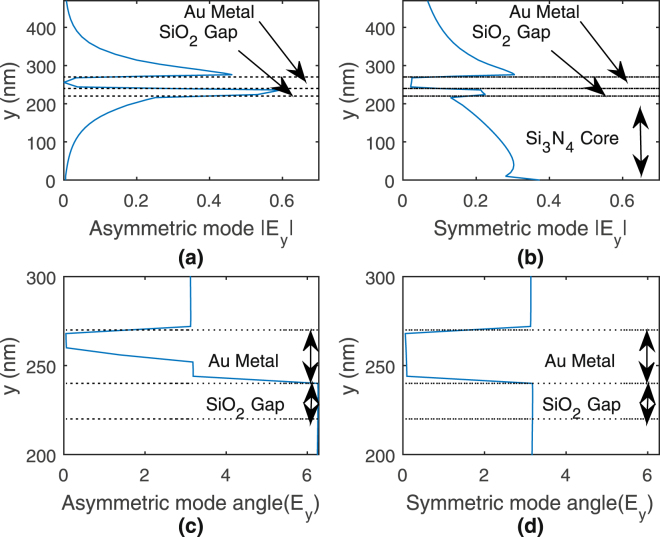
Plots of (**a**,**b**) magnitude and (**c**,**d**) phase of asymmetric and symmetric modes’ transverse field *E*_*y*_ along the waveguide height (*y* direction) at the taper tip and at *x* = 0.

**Figure 3:**
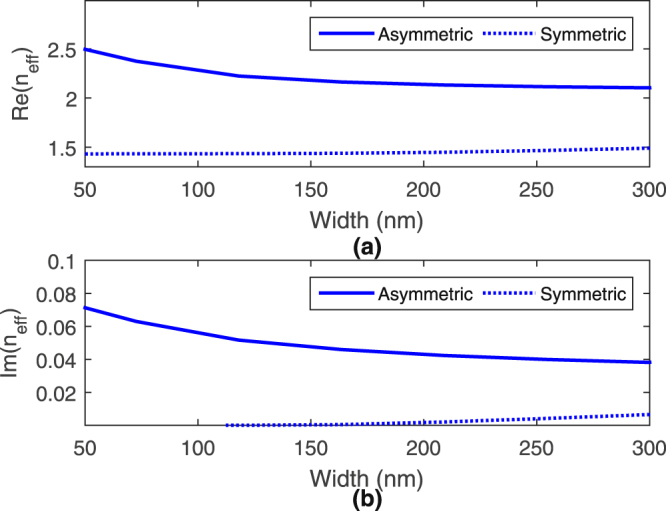
Plots of (**a**) real and (**b**) imaginary parts of effective index of asymmetric and symmetric mode for different waveguide widths.

The proposed tapered-coupler generally supports 2 hybrid supermodes along the *z* propagation direction with fields partially in the core and on the metal surface. These 2 supermodes are termed as symmetric and asymmetric modes, due to the characteristic phase shifts of 0 and *π* in the transverse field *E*_*y*_, respectively, across the metal layer. At smaller widths, the symmetric and asymmetric modes approach the well-known long- and short-range surface plasmon (SRSP) modes of a DMD waveguide, respectively^24^. Figure 2(a) and (b) plot the magnitude of the asymmetric and symmetric modes’ transverse field *E*_*y*_ along the waveguide height (*y* direction) at the taper tip and at *x* = 0. As can be seen, the asymmetric mode has transverse field strongly confined at the metal surface (stronger exponential decay) while the symmetric mode has significant field leaking into the core and top cladding. Figure 2(c) and (d) plot the corresponding phase of the asymmetric and symmetric modes’ transverse field *E*_*y*_. Figure 2(c) shows a *π* phase shift of the asymmetric mode’s transverse field within the metal layer while Fig. 2(d) shows a 0 degree phase shift of the symmetric mode’s transverse field within the metal layer. The asymmetric mode thus has a *π* phase shift in it’s transverse field across the metal layer and it is the reason for naming it “asymmetric”. Figure 3(a) and (b) plots the real and imaginary parts of the effective index of symmetric and asymmetric modes for different waveguide widths (or length of the tapered hybrid waveguide structure). As can be deduced, when the width reduces, the real part of the asymmetric mode’s effective index increases, indicating more field confinement towards the metal interface and hence greater metal dissipation, as reflected in the corresponding increase in the imaginary part of the effective index^25^. On the other hand, for the symmetric mode, the real part of the effective index reduces towards the background index as width reduces, indicating more field leakage into the surrounding media and lower metal dissipation, as noted by a decrease in imaginary part of the effective index. Figure 3 shows similar characteristics as the corresponding long- and SRSP modes^24^.

**Figure 4:**
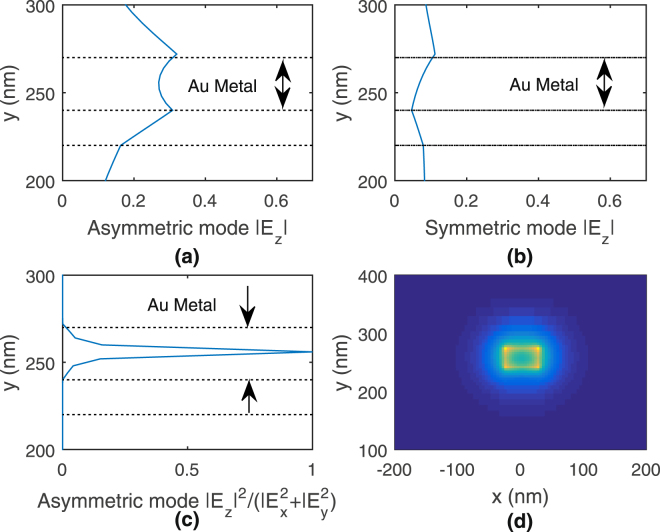
Plots of (**a**) asymmetric and (**b**) symmetric mode longitudinal field *E*_*z*_ magnitude, (**c**) normalized asymmetric mode $\frac{|{E}_{z}{|}^{2}}{|{E}_{x}{|}^{2}+|{E}_{y}{|}^{2}}$ and (**d**) asymmetric mode longitudinal *E*_*z*_ magnitude across *xy* cross-section.

Considering the longitudinal mode component *E*_*z*_, it can be seen from Fig. 4(a,b) that the asymmetric mode has a stronger and more even-distributed longitudinal component across the metal layer, compared to the symmetric mode. In fact from Fig. 4(c), we can see that the asymmetric mode’s longitudinal component dominates within the metal, especially at the centre of the metal^26^. It was previously discussed in^27^ that the longitudinal field at the tip of a DMD waveguide structure in fact facilitates better transfer of optical energy instead of a capacitive type field (fringing fields) at the tip of a metal-dielectric-metal waveguide structure^28^. From the modal analysis, we deduce that the asymmetric mode would be a more suitable mode, particularly due to its stronger confinement of field and its more uniform and stronger distribution of longitudinal electric field (see Fig. 4(d)), which would be the field component at the tip responsible for strong field enhancements. The wavelength for modal analysis is 771 nm.

### Coupling Analysis

**Figure 5:**
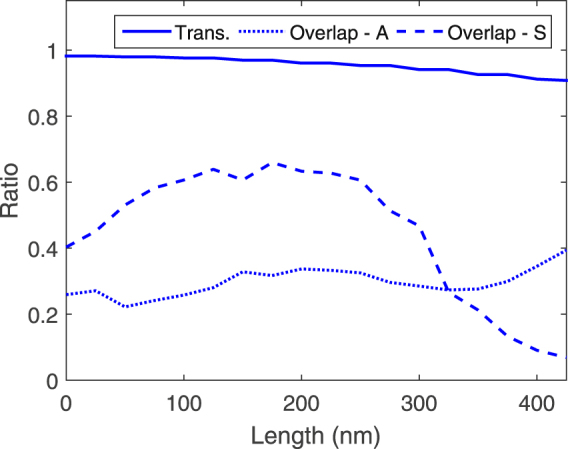
Plot of transmission efficiency and overlaps with the symmetric (Overlap-S) and asymmetric (Overlap-A) modes.

In the previous section, we deduced that the asymmetric mode is the mode of interest. In this section, we analyze the coupling efficiencies of both asymmetric and symmetric modes within the tapered-coupler. For this coupling analysis, the tapered-coupler tip is assumed to be terminated by an infinitely long plasmonic waveguide of width *W*_*t*_ = 50 nm. Various *xy* monitor planes are placed along the tapered-coupler and steady-state field values are determined after the simulation ends. The transmission is then calculated from these monitor planes by taking the ratio of the transmitted power $\frac{1}{2}\Re \{\int {\bf{P}}({\boldsymbol{\omega }})\cdot \hat{z}dS\}$ to the source power. The Poynting component in the *z* direction is calculated from the transverse fields on the monitor plane at every grid point and the integration of the individual Poynting components at every grid point, over the entire monitor plane, gives the transmitted power. Figure 5 plots the transmission efficiency along with the overlaps with the symmetric and asymmetric modes across the length of the tapered-coupler. As can be seen from the figure, the transmission efficiency into and out of the coupler is 98% and 90%, respectively. Of the power transmitted into the coupler, the coupling efficiency into the symmetric and asymmetric modes are 40% and 26%, respectively. Along the taper, we see that the asymmetric mode generally maintains consistent coupling efficiency and this efficiency increases slightly towards the taper tip. The coupling efficiency of the asymmetric mode at the taper tip is 39.3%. On the other hand, we see that the symmetric mode has coupling efficiency that increases slightly towards the centre of the tapered-coupler (which could possibly be due to inter-modal coupling effects^2^) but then decreases towards the tip, due to leakage into radiation. The mode is thus primarily asymmetric at the taper tip, with a consistent coupling efficiency of 39.3%.

### Resonance Analysis

**Figure 6:**
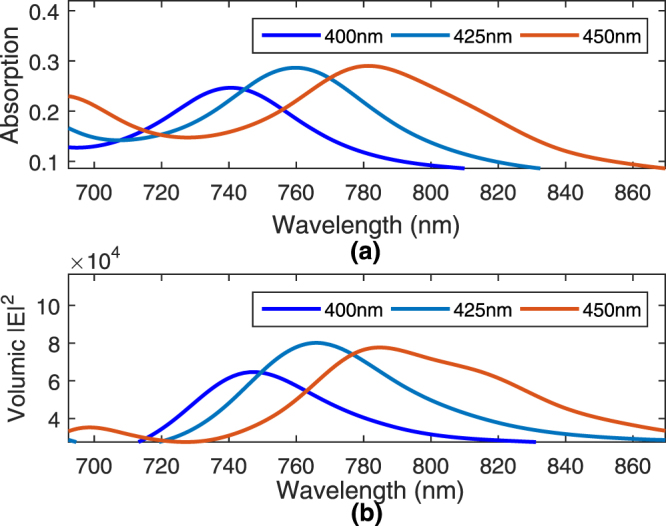
Plots of (**a**) absorption and (**b**) integrated electric field over volume region around taper tip, for the tapered-coupler structure.

**Figure 7:**
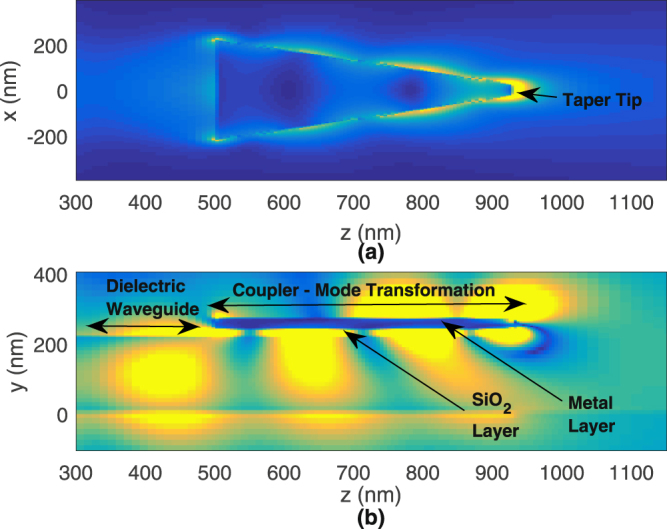
Plots of the (**a**) electric field magnitude at the top *xz* plane with *y* = 255 nm (centre of the metal layer) and (**b**) transverse electric field |*E*_*y*_| at the *yz* plane with *x* = 0 nm, along the length of the tapered-coupler.

In the previous section, we analyzed the coupling efficiencies of the asymmetric and symmetric modes and noted that the symmetric mode leaks into radiation towards the tapered tip, while the asymmetric mode converts into the well-known SRSP mode with coupling efficiency of 39.3% at the taper tip. In this section, we analyze the resonance property of the proposed tapered-coupler that is terminated abruptly, as shown in Fig. 1. The resonance property is analyzed from the wavelength dependent absorption within the metal layer. This absorption is determined by first calculating the power absorbed using the following formula

1 $${P}_{abs}=\frac{1}{2}{\int }_{v}\,\omega {\varepsilon }_{metal}^{I}{\varepsilon }_{0}|E{|}^{2}dv,$$

in which ${\varepsilon }_{metal}^{I}$ is the imaginary part of the relative electric permittivity of gold (Au) and |***E***|^2^ is the field intensity within the metal layer. The integration is taken over the entire metal layer. The absorption is then deduced by dividing the power absorbed with the source power. This absorption is plotted in Fig. 6(a) for tapered-couplers of varying lengths of 400, 425 and 450 nm. From the figure, we see that the peak absorption resonance shifts towards longer wavelengths as the length increases. For the designed length of 425 nm, the maximum absorption occurs at about 760 nm. Figure 6(b) plots the total electric field intensity within a volume of region around the taper tip. It can be seen that the field intensity increases to as high as 80000 at wavelength of 767 nm, for the designed length of 425 nm. A similar trend is noted where the wavelength corresponding to the maximum intensity shifts towards longer wavelengths as the length increases. For the case of designed length of 425 nm, Fig. 7(a) shows the electric field magnitude on the top *xz* plane with *y* = 255 nm. This is on the plane slicing the centre of the metal layer. Here we can see strong localized fields at the tip of the taper. Figure 7(b) shows the corresponding transverse electric field |*E*_*y*_| along the *yz* plane with *x* = 0 nm. This is seeing from the side of the tapered-coupler. Here, we can see the input dielectric waveguide mode propagating towards the input of the tapered-coupler that is at *z* = 500 nm. Along the tapered-coupler, from *z* = 500 nm to *z* = 925 nm, we observe the dielectric waveguide mode being pulled upwards, towards the metal layer. Localized fields are then formed at the tip of the metal layer.

## Discussion

**Figure 8:**
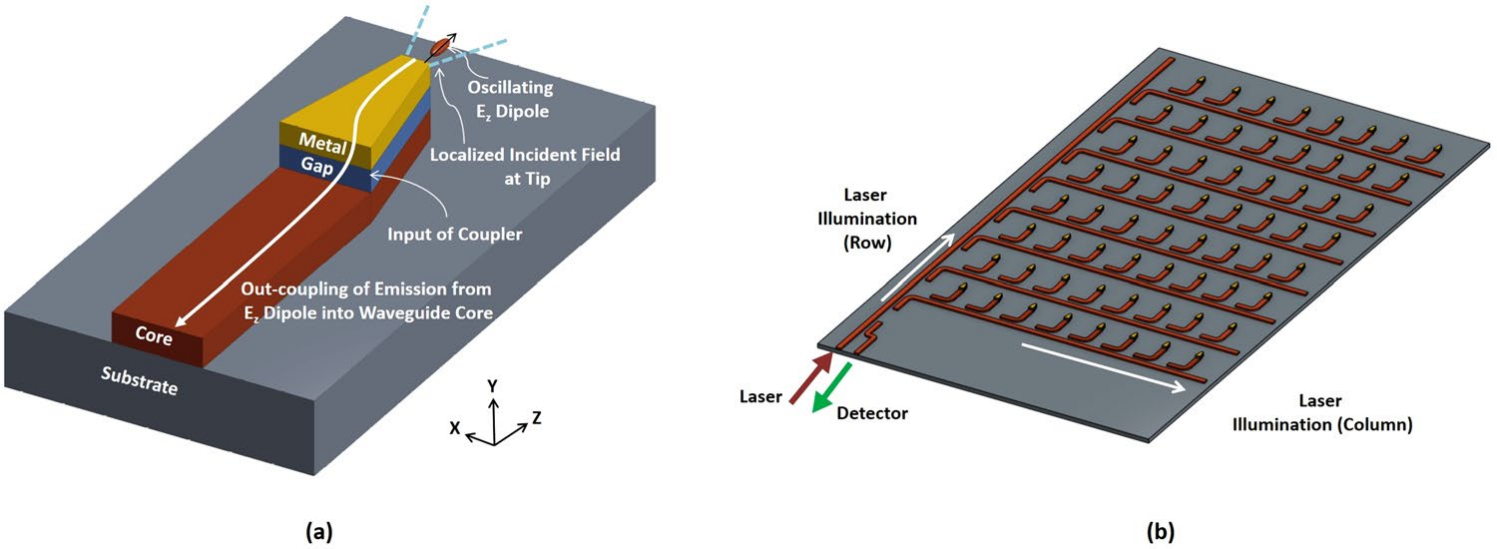
Schematic of (**a**) out-coupling of emission from *E*_*z*_ dipole into waveguide core and (**b**) 8 × 8 array of single-element tapered-couplers.

We presented a design of a tapered-coupler on a CMOS-compatible *Si*_3_ *N*_4_ platform that is able to couple optical energy from a *Si*_3_ *N*_4_ core waveguide to a plasmonic tip of nanometer scale. Such proposed tapered-coupler can find significant applications as plasmonic elements that provide localized field enhancements to enhance Raman and fluorescence emissions in SERS and MEF, respectively. Compared to conventional metal plasmonic structures in SERS and MEF, such as gold nanoparticles^29^ or planar substrate designs, the proposed tapered-coupler can be properly excited from integrated on-chip sources such as hybrid lasers^30^, thus allowing for development of much-more compact and low-cost bio-sensors - moving away from traditional free-space based illumination methods. In addition, for a SERS biosensor, one of the most critical components is the plasmonic substrate onto which the analyte is absorbed. It is imperative that the plasmonic platform should have reproducible and repeatable signal enhancement at all locations, which is critical when intensity based sensors are being developed. By using an array of tapered-coupler structure such as in Fig. 8(b), it is possible to move towards a solution where the plasmonic platform is not only able to couple light from an on-chip laser (such as through hybrid integration of lasers, see^30^) but also able to reproduce and repeat signal enhancement at all locations.

**Figure 9:**
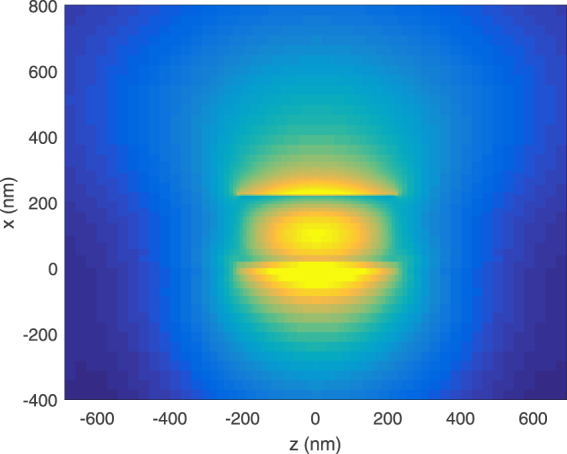
Plot of out-coupled |*E*_*y*_| field distribution across the *Si*_3_ *N*_4_ core waveguide for a *E*_*z*_ dipole oscillating 5 nm away from the taper tip.

One of the important figure of merits in SERS is the field enhancement factor, which can be expressed as a product of the local field enhancement due to incident excitation and the radiation enhancement or the Purcell factor^31–34^. For the proposed tapered-coupler with a tip width of 50 nm, the local field enhancement at a point 5 nm away from the tip is 59. This local field enhancement is calculated by taking the ratio between the steady-state electric field intensity at the same point (for a fundamental TM mode excitation) to the steady-state electric-field intensity within the *Si*_3_ *N*_4_ waveguide (assuming it is a straight waveguide). The Purcell factor of an *E*_*z*_ dipole placed at this same point is calculated as 233. The Purcell factor is calculated as the ratio of the emitted dipole power in the presence of the tapered-coupler to the emitted dipole power in a homogeneous environment without the presence of the tapered-coupler. The total enhancement is thus 13747, which is the product of the local field enhancement and the Purcell factor. This total enhancement factor can be increased by reducing tip widths to, for example, 20 nm. The local field enhancement, Purcell factor and total field enhancement for a tip width of 20 nm are 720, 1709 and 1.23 × 10^6^, respectively. These calculations are made at a wavelength of 770 nm. The proposed tapered-coupler structure can also potentially allow for out-coupling of Raman emissions through the waveguide structure, as illustrated in Fig. 8(a), where the *E*_*z*_ dipole oscillates and couples light back into the waveguide core. For a tapered-coupler with tip width of 50 nm and with an *E*_*z*_ dipole oscillating 5 nm away from the taper tip, the coupling efficiency back into the *Si*_3_ *N*_4_ waveguide is calculated as 4.32%. This coupling efficiency was calculated by taking the ratio of the *z*-directed Poynting power (calculated by integrating *z*-directed Poynting power components across a *xy* plane through the *Si*_3_ *N*_4_ waveguide core) to the emitted dipole power. Figure 9 plots the |*E*_*y*_| field distribution across the *Si*_3_ *N*_4_ waveguide for a *E*_*z*_ dipole oscillating 5 nm away from the taper tip. As can be deduced, this field distribution corresponds to the fundamental TM mode of the *Si*_3_ *N*_4_ waveguide.

## Conclusion

In this report, a tapered-coupler structure was proposed on an integrated-photonic platform based on silicon nitride to couple optical energy from a dielectric waveguide to a plasmonic tip. Mode analysis showed that the tapered-coupler is able to support the required asymmetric plasmonic mode that has strong localized fields at the metal surface. This asymmetric plasmonic mode approaches the well-known short-range surface plasmon mode at smaller widths. The coupling efficiency of the asymmetric plasmonic mode along the proposed tapered-coupler structure was shown to be generally consistent and, at the taper tip of width 50 nm, the coupling efficiency was shown to be 39.3%. Resonance analysis showed that the tapered-coupler has a plasmonic absorption resonance peak at 760 nm and the peak electric field intensity is at 767 nm, which is close to the laser excitation sources for biosensing applications. A potential application of the tapered-coupler structure for SERS based biosensing was discussed, in which, field enhancement factors as high as 1.23 × 10^6^ were deduced for taper tip width as small as 20 nm. The proposed tapered-coupler sets up interesting possibilities towards moving to an all-integrated on-chip SERS based bio-sensor platform - away from traditional free-space based illumination platforms.

## Methods

The theoretical electromagnetic wave analysis and design framework were performed with the help of full three-dimensional finite-difference time-domain (FDTD) method as well as an eigenmode solver (Lumerical FDTD, Lumerical Solutions Inc.). In the FDTD method, a non-uniform mesh scheme with minimum mesh step sizes of 0.25 nm, was used. Perfectly matched layers were used as absorbing boundary conditions. Overlap integrals were performed using the following equation

2 $$Overlap=\Re [\frac{(\int {\overrightarrow{E}}_{1}\times {{\overrightarrow{H}}_{2}}^{\ast }\cdot \overrightarrow{d}S)(\int {\overrightarrow{{\rm{E}}}}_{2}\times {{\overrightarrow{H}}_{1}}^{\ast }\cdot \overrightarrow{d}S)}{\int {\overrightarrow{E}}_{1}\times {{\overrightarrow{H}}_{1}}^{\ast }\cdot \overrightarrow{d}S}]\frac{1}{\Re (\int {\overrightarrow{E}}_{2}\times {{\overrightarrow{H}}_{2}}^{\ast }\cdot \overrightarrow{d}S)}$$
